# Crop classification in high-resolution remote sensing images based on multi-scale feature fusion semantic segmentation model

**DOI:** 10.3389/fpls.2023.1196634

**Published:** 2023-08-01

**Authors:** Tingyu Lu, Meixiang Gao, Lei Wang

**Affiliations:** ^1^ College of Geographical Sciences, Harbin Normal University, Harbin, China; ^2^ Department of Geography and Spatial Information Techniques, Ningbo University, Ningbo, China; ^3^ School of Civil and Environmental Engineering and Geography Science, Ningbo University, Ningbo, China; ^4^ Department of Surveying Engineering, Heilongjiang Institute of Technology, Harbin, China

**Keywords:** remote sensing, crop classification, deep learning, convolutional neural network, multi-scale feature

## Abstract

The great success of deep learning in the field of computer vision provides a development opportunity for intelligent information extraction of remote sensing images. In the field of agriculture, a large number of deep convolutional neural networks have been applied to crop spatial distribution recognition. In this paper, crop mapping is defined as a semantic segmentation problem, and a multi-scale feature fusion semantic segmentation model MSSNet is proposed for crop recognition, aiming at the key problem that multi-scale neural networks can learn multiple features under different sensitivity fields to improve classification accuracy and fine-grained image classification. Firstly, the network uses multi-branch asymmetric convolution and dilated convolution. Each branch concatenates conventional convolution with convolution nuclei of different sizes with dilated convolution with different expansion coefficients. Then, the features extracted from each branch are spliced to achieve multi-scale feature fusion. Finally, a skip connection is used to combine low-level features from the shallow network with abstract features from the deep network to further enrich the semantic information. In the experiment of crop classification using Sentinel-2 remote sensing image, it was found that the method made full use of spectral and spatial characteristics of crop, achieved good recognition effect. The output crop classification mapping was better in plot segmentation and edge characterization of ground objects. This study can provide a good reference for high-precision crop mapping and field plot extraction, and at the same time, avoid excessive data acquisition and processing.

## 1 Introduction

With the rapid development of remote sensing technology, the quality and updating speed of remote sensing data have been significantly improved, and multi-source remote sensing data has been widely applied in agriculture, forestry, Marine, environmental protection and other fields (Sun, 2020). Remote sensing image classification has always been a very active research topic in the application of remote sensing technology, which refers to the use of remote sensing data to make land use or land cover maps (Luo, 2011).

At present, the application based on artificial intelligence model and algorithm has become very common. Machine learning and deep learning are the methods to realize artificial intelligence. With the continuous innovation of deep learning, the field of computer vision has developed rapidly in the past few years and made breakthroughs constantly (Hopfield, 1982; Bengio and Delalleau, 2011). The development of computer vision is driven by the innovation of algorithms, the increase in the amount of visual data and the improvement of computing power. In image classification, target detection and location, image segmentation and other tasks, deep learning algorithms surpass traditional statistical methods on a large number of benchmarks, and even exceed human beings in image and target recognition (Bengio, 2009; LeCun et al., 2015).

In the field of agriculture, using remote sensing data to classify crops is an important research content. Timely and accurate acquisition of spatial distribution and planting area of crops by utilizing spatio-temporal scale advantages of remote sensing images is of great significance for ensuring food security and promoting sustainable agricultural development (Kussul et al., 2017). High resolution remote sensing image has the characteristics of high background complexity, rich detail information and diversified spatial structure, so the classification accuracy is often low when the traditional machine learning classification algorithm is applied to the classification of high resolution remote sensing image. In recent years, many researchers have tried to build semantic segmentation network through deep learning algorithm and applied it in pixel-level ground object fine classification. Remote sensing image classification based on artificial neural network has become a development trend (Zhong et al., 2018; Rustowicz et al., 2019).

For traditional machine learning models and popular deep learning models, the architecture design of the model itself and super-parameter fine-tuning determine the feature extraction capability of the model, and the strength of the feature extraction capability is a decisive factor affecting the model performance. The high efficiency of deep learning algorithm is reflected in its independent dependence on highly complex feature engineering, and its high performance is reflected in its powerful feature extraction ability. Therefore, how to enhance the feature extraction ability of the algorithm is the essential problem of deep learning model architecture design (Kawaguchi et al., 2017).

Multi-scale refers to the sampling processing of signals with different granularity. In deep learning algorithm, it means that the model learns different features at different scales, such as fine features and rough features, as well as the combination of the two features. This method has been proved to effectively improve the performance of the model. The idea of multi-scale feature fusion technology is to extract image features under different sensory fields. At present, there are mainly two types of multi-scale feature network design paradigms, one is skip connection architecture based on deep convolutional neural network (DCNN), such as UNet, VNet (Milletari et al., 2016), FCN series (Long et al., 2015), RefineNet (Lin et al., 2017), etc. This kind of network is characterized by the use of pre-training weights or DCNN (represented by residual network) in the coding stage, and the acceptance of low-level features through skip connections in the decoding stage, and the fusion of low-level and abstract features, so as to achieve multi-scale feature extraction. The other type adopts parallel multi-branch structure design, such as PSPNet (Zhao et al., 2017), GoogleNet, DeepLab series (Chen et al., 2017; Chen et al., 2018; Chen et al., 2018), etc., which is characterized by using hollow convolution or convolution kernel of various sizes to extract features from different receptor fields, and finally merging multiple channels to form multi-scale features.

Multi-scale feature fusion network is widely used in computer vision tasks such as target detection and image classification. Varadarajan et al. (2021) designed an object detection network composed of 22 convolution layers. By using multi-scale feature fusion technology, the network can well identify objects of different sizes and shapes from images. Sang et al. (2022) proposed a target tracking network MTTNet based on multi-scale global retrieval and spatial-temporal consistency matching, and used spatial pyramid pool to solve the problem of multi-scale feature extraction. The experimental results show that the network has stable performance and can effectively perform long-term target tracking tasks. Wu et al. (2022) integrated the hierarchical pyramid pooling module into the full convolutional neural network, and the improved network was able to collect multi-scale context information. The good performance of the network was verified in the robot object grabbing experiment. In the study of fine-grained image classification, Liu et al (Liu et al., 2021). fused the attention module with multi-scale feature expression in order to distinguish the subtle differences between the subcategories of the main category. The improved network can learn a list of accurate feature maps. Xie et al. (0000) proposed a multi-scale densely connected convolutional neural network MS-DenseNet when studying hyperspectral image classification. By learning multi-scale patches around each pixel, they made full use of multi-scale information. Wang et al. (2022) proposed a multi-scale convolutional neural network point cloud filtering algorithm based on attention mechanism to solve the problem of low accuracy of traditional filtering methods when processing lidar data, combining channel and spatial attention module with multi-scale convolution kernel. The lidar point cloud feature maps output by the algorithm at different scales can adjust the weights of each channel layer and different spatial regions adaptively, so that the network pays extra attention to important information, thus improving the classification performance of the model.

In recent years, many deep semantic segmentation networks using multi-scale feature fusion methods have been applied to pixel level classification of remote sensing images or scene classification of remote sensing images. A large number of research results show that it is an effective method to obtain better image classification results. When conducting large-scale land cover classification, Gao et al. (2021) found that the traditional sliding window convolutional neural network has a large computational overhead and the classification results are not precise enough. To solve this problem, a new object-oriented deep learning framework was proposed, which uses residual networks to learn features on different adjacent scales and achieves a balance between weak semantics and strong features. When studying the classification of complex remote sensing scenes, Bi et al. (2021) found that the complex spatial arrangement and object size changes in large-scale aerial images were challenging for classification models. In order to enhance the feature expression ability of remote sensing scenes, a multi-scale expanded convolution operator was designed. To solve the “small sample” problem of hyperspectral image classification, Gong et al. (2021) designed a lightweight multi-scale attention pyramid pooling network MSPN, whose core components included a multi-scale three-dimensional CNN module and a squeezing excitation attention module. The network learns and fuses deeper spatial spectral features with fewer training samples, and verifies MSPN’s good performance on publicly available hyperspectral data sets. Liao et al. (2022) developed multi-scale object-driven convolutional neural network multi-OCNNs, which can capture the depth and context information contained in the reference samples, and has achieved good results in land cover classification based on multi-source high-resolution images such as SPOT-6, Gaofen-2 and ALOS.

In summary, a large number of deep learning models represented by convolutional neural networks have been applied to intelligent information extraction tasks of remote sensing images. However, there are few researches on exploiting the potential of multi-spectral remote sensing in crop mapping by using multi-scale feature fusion semantic segmentation model. Models that use pre-trained networks combined with multiple jump connections to achieve feature fusion tend to be deeper, resulting in larger model parameters and a significant increase in computational overhead. Skipping connections can only alleviate the problem of single feature size to a certain extent, and the requirements of fine-grained segmentation cannot be met in the initial stage of training, resulting in insufficient segmentation results. In order to solve this problem, this paper proposes a semantic segmentation model with residual network as the backbone and receiving field module for multi-scale and multi-scale feature fusion. The performance of the model was evaluated in the experiment of crop classification based on Sentinel-2 high-resolution remote sensing image.

## 2 Multi-scale feature extraction method based on deep convolutional neural network

On the premise of effectively alleviating the problem of gradient disappearance, the residuals network (ResNet) improves the performance of the model by adding considerable depth. In addition to the common residuals network of 18 layers, 34 layers and 50 layers, there are ResNet-101 and ResNet-152 at a deeper level. A modest increase in the depth of the network is beneficial to the performance of the model, and a large number of experiments have shown that changing the width of the network can achieve the same purpose. Multi-scale feature extraction modules based on parallel multi-branch structure design have been proposed one after another. In this chapter, several important convolutional modules are described in detail.

### 2.1 Inception

**Figure 1:**
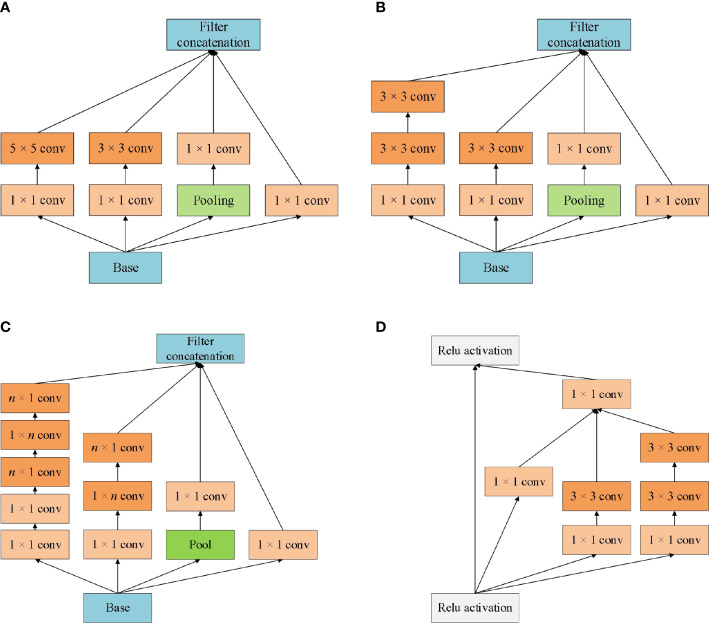
Different versions of the Inception module architecture schematic. **(A)** Inception V1; **(B)** Inception V2; **(C)** Inception V3; **(D)** Inception V4.

The design concept of Inception is to use convolution kernels of different sizes to realize the perception of multi-level features, and finally fusion to obtain better representation of images. Inception module is the core component of the GoogleNet network. From Inception V1 to Inception V4, each version is the optimization of the previous version, with the number of parameters decreasing and the running speed and accuracy gradually improving. The different versions of the Inception model structure are shown in **Figure 1**.

**Figure 2:**
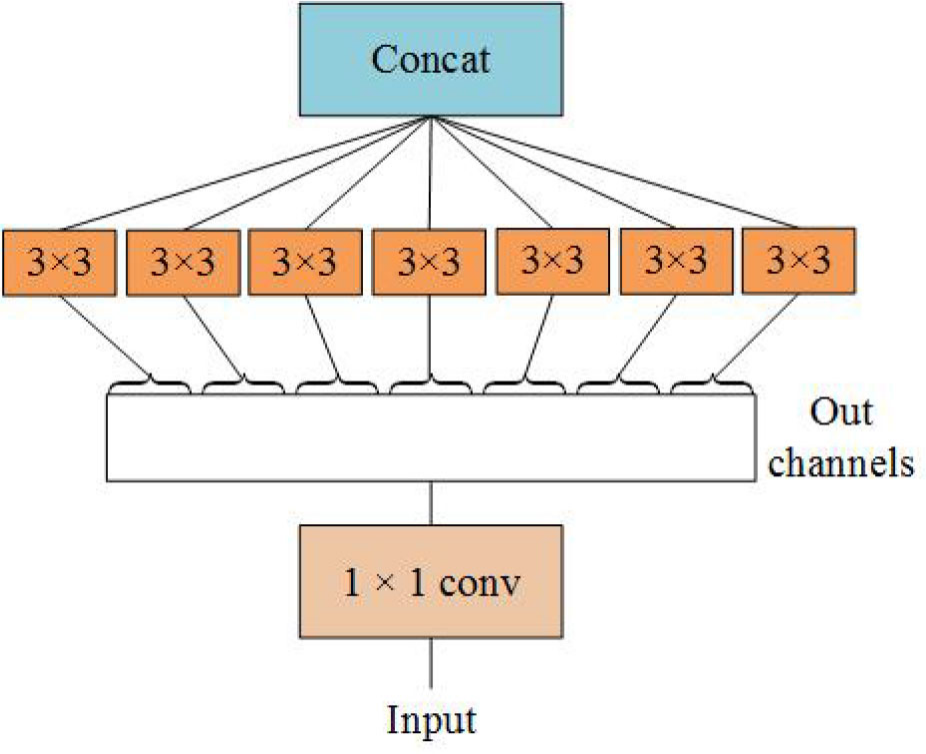
Perform the 3 × 3 convolution on each channel of the 1×1 convolution.

Inception module realizes multi-scale feature space superposition through parallel multi-branch operations and uses intensive operations to maintain model sparsity. Xception to Inception - V3 is improved, and put forward the depth of Separable convolution (Depthwise Separable Convolutions), Inception - V3 will channel is divided into four groups respectively carry out 1 x 1 convolution computation, Xception performs a 1 × 1 convolution calculation for each channel’s feature graph and concatenates the feature forces. Completely decouple channel and spatial dependencies. Xception has the same number of parameters as Inception-V3, but with better performance and more efficient use of network parameters, as shown in **Figure 2** for its structure.

### 2.2 Atrous spatial pyramid pooling

**Figure 3:**
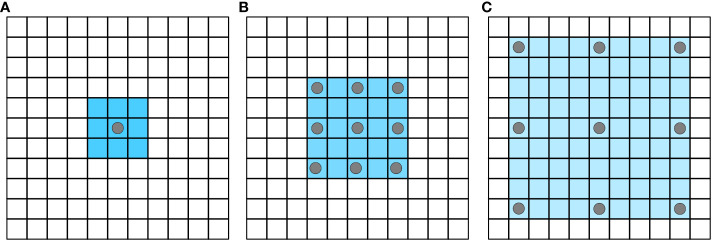
Atrous convolution increases the receptive field without losing information. **(A)** atrous_rate=1; **(B)** atrous_rate=2; **(C)** atrous_rate=4.

Atrous Spatial Pyramid Pooling (ASPP) uses dilated convolution with different dilation coefficients to extract multi-scale features. Dilated convolutions (Yu and Koltun, 2016) add dilation into the standard convolutions to increase the field of perception (See **Figure 3**).

The calculation process of dilated convolution is shown in the following formula, where *H_in_* and *H_out_* respectively represent the height of the input and output feature graphs, and *W_in_* and *W_out_* respectively represent the width of the input and output feature graphs.


(1) $$H_{out}=\frac{H_{in}+2\times \text{padding}[0]-\text{atrous}[0]\times \left(\text{kernel}[0]-1\right)-1}{stride[0]}+1$$

(2) $$W_{out}=\frac{W_{in}+2\times \text{padding}[1]-\text{atrous}[1]\times \left(\text{kernel}[1]-1\right)-1}{stride[1]}+1$$

Some semantic segmentation algorithms based on full convolutional neural networks, such as FCN-8S and FCN-16S, need continuous up-sampling in order to achieve the same resolution of input and output images. However, this process cannot recover the loss of detail information caused by previous pooling. Dilated convolution can reduce such loss to a certain extent.

**Figure 4:**
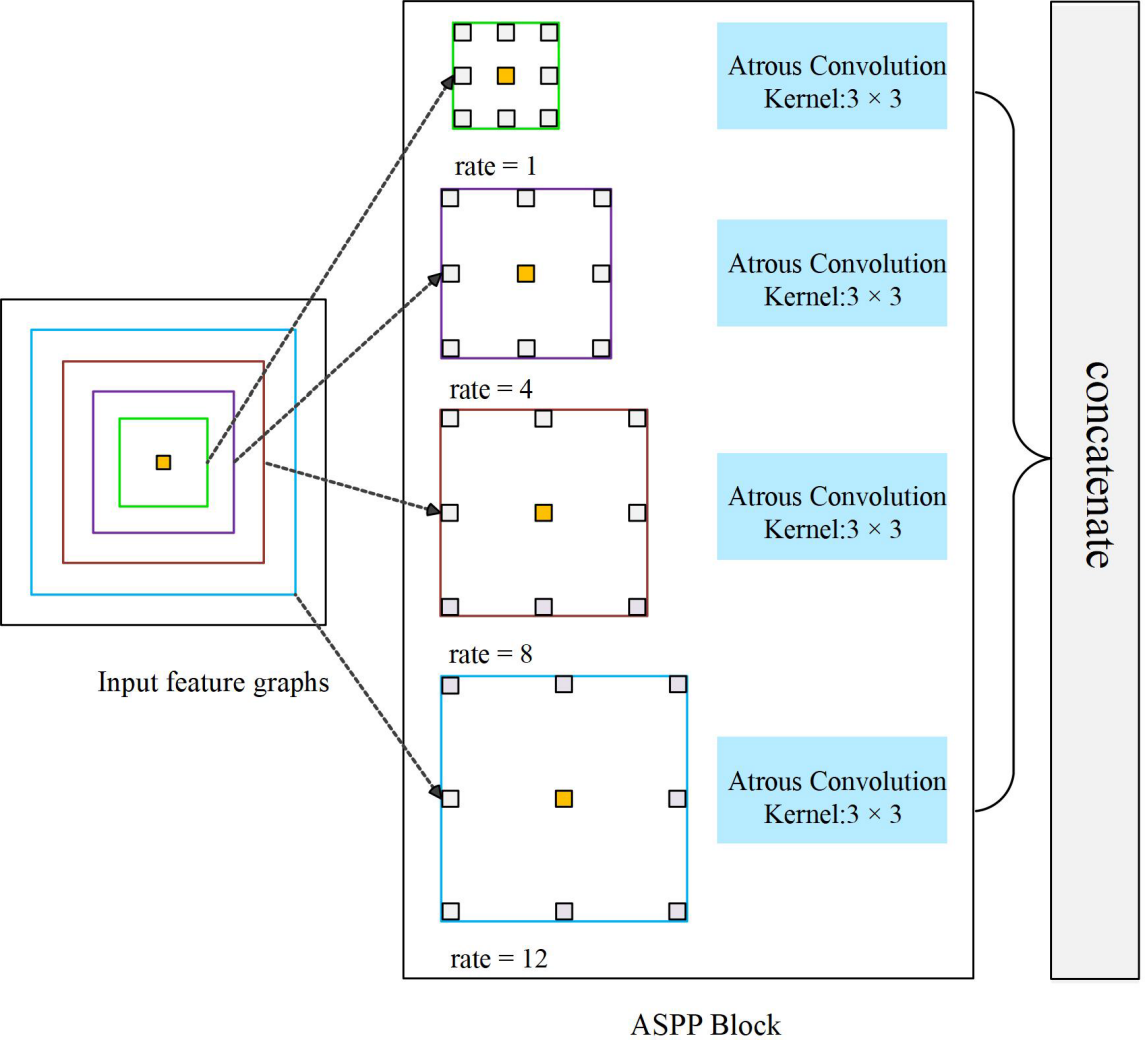
An ASPP Block capable of extracting four scale features.

ASPP module first appeared in the semantic segmentation algorithm DeepLab V2, consisting of 3 × 3 convolution of four different expansion coefficients. Subsequently, ASPP was applied to many image classifications tasks as an independent module (Yuan et al., 2019; Liu et al., 2021; Pedrayes et al., 2021). The branch structure inside ASPP is not invariable, and designers often adopt different parameter configurations according to different application scenarios. **Figure 4** shows an ASPP Block containing four branches. By setting four different dilation, the module is capable of feature extraction from four different scales.

ASPP uses filters with multiple sampling rates and effective field of view to detect the incoming convolutional feature map, so as to capture objects and image context information at multiple scales, keep image resolution unchanged, obtain more intensive feature response, and better restore the details of the original image.

### 2.3 Receptive field block

**Figure 5:**
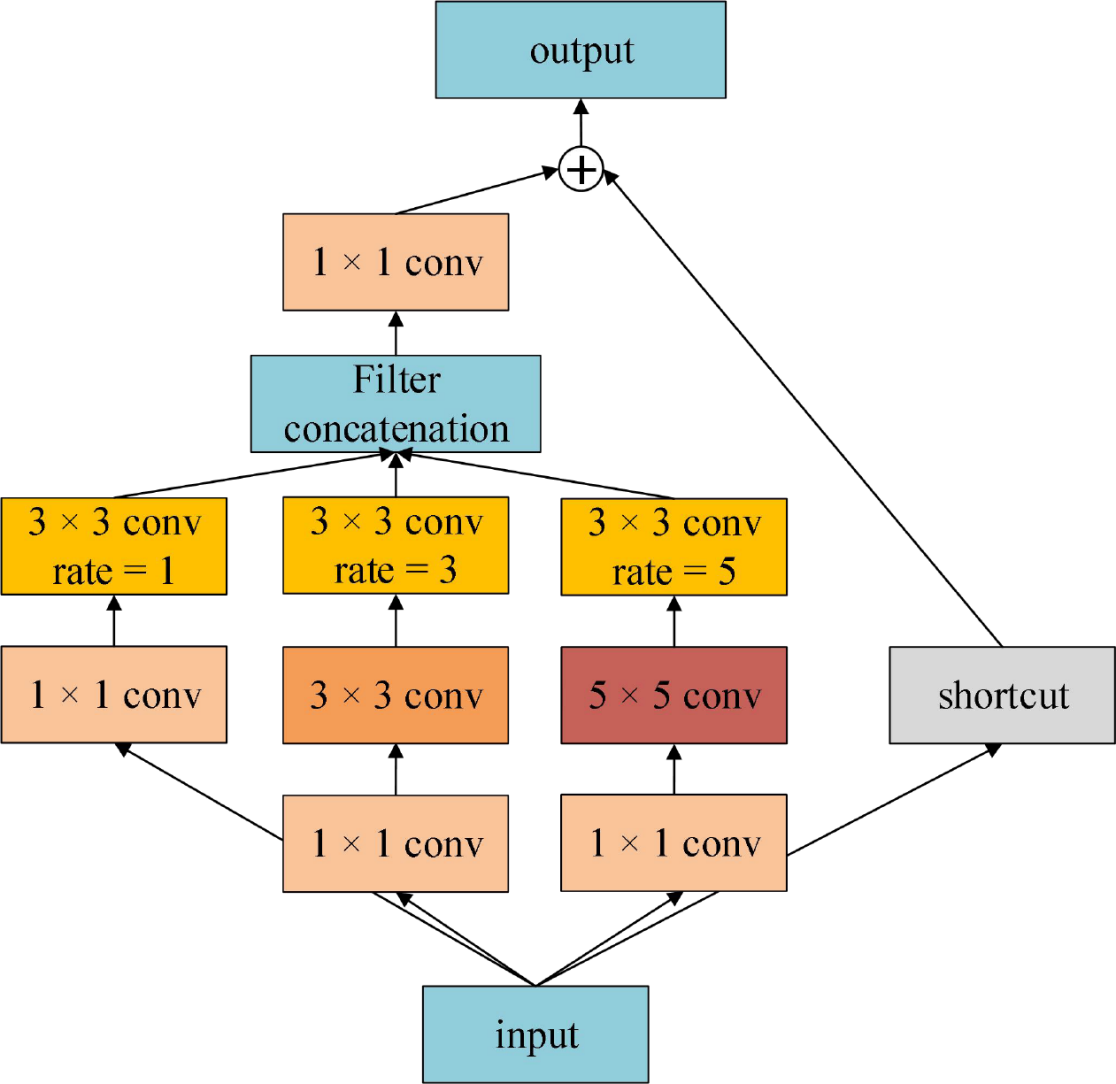
The architecture of RFB.

Receptive Field Block (RFB) refers to the design concept of Inception. In each branch structure, conventional convolution of convolution kernel of specific size is first used, then dilated convolution is added, and multi-scale features are extracted by group convolution. Dilated convolution increases receptive field. The RFB module considers the relationship between the receptive field center and the target region to enhance the feature recognition and robustness (Liu et al., 2018). The structure of RFB is shown in **Figure 5**. The extracted multi-scale features are fused and input to the next layer by adding 1 × 1 convolution and identity shortcut connection. RFB is a lightweight feature extraction module, which can be conveniently configured in convolutional neural networks. Especially in some target detection tasks, RFB has brought significant performance gains to detection networks (Li et al., 2019; Liu et al., 2019; Yuan et al., 2021).

## 3 Multi-scale feature fusion network-MSSNet

### 3.1 Study area

**Figure 6:**
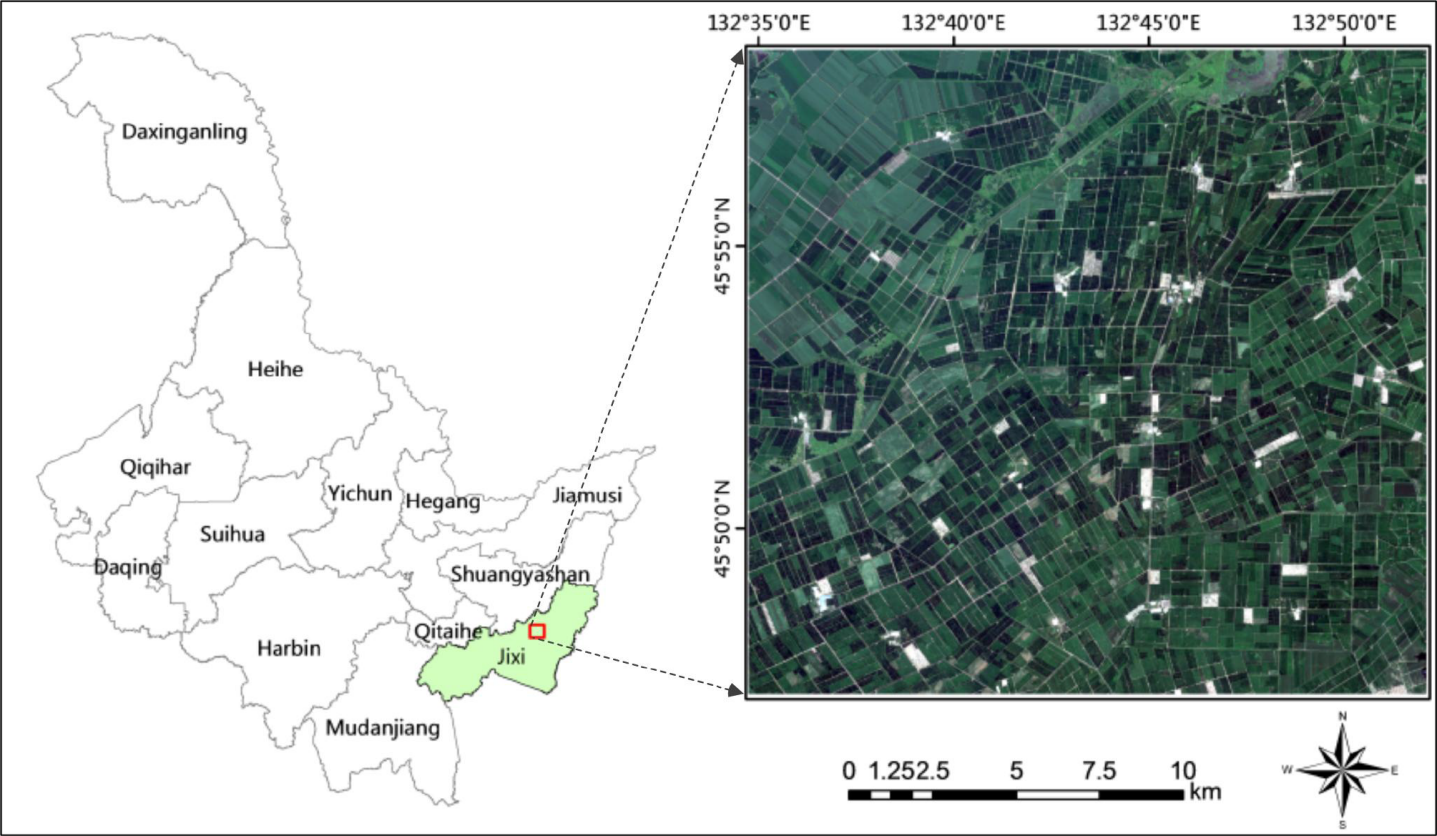
Study area with its RGB image composite derived from Sentinel-2 imagery.

The study area is Yunshan Farm and 850 Farm located in Hulin City, Heilongjiang Province. The longitude range is 132°35 ‘21 “~132°51’ 46” E, and the latitude range is 45°47 ‘21 “~45°58’ 11” N. Located in the famous Sanjiang Plain, this area is a temperate continental monsoon climate with an effective accumulated temperature of 2501°C. 80% of the cultivated land is low-wet land with fertile soil and abundant water resources. It mainly grows corn, rice and soybeans, and is an important commercial grain base in China. The location and scope of the research area are shown in **Figure 6**.

The remote sensing data uses the high-resolution multi-spectral image of Sentinel-2 satellite developed by ESA. Sentinel-2 is divided into 2A and 2B satellites with a revisit period of 5 days. Sentinel-2 can cover 13 spectral bands and provide multi-spectral images with spatial resolution of 10 meters, 20 meters and 60 meters (Verrelst et al., 2012). Widely used in agricultural resources monitoring and crop yield estimation, geological survey, land use dynamic monitoring and other fields. Sentinel-2 remote sensing data used in this paper is Level-1C data that was imaged on July 28, 2020, and is derived from Sentinel Hub. Sen2cor 2.11 is used to preprocess the data. Firstly, radiometric calibration and atmospheric correction were carried out for multi-spectral images to eliminate radiation errors caused by atmospheric scattering, etc. Then SNAP 9.0 and ENVI 5.3 platforms were used to generate reflectance Level 2A data at the bottom of the atmosphere. Finally, the resolution of the resampling remote sensing image was 10 meters, and the study area was about 423 square kilometers.

### 3.2 Features extraction and training set construction

**Figure 7:**
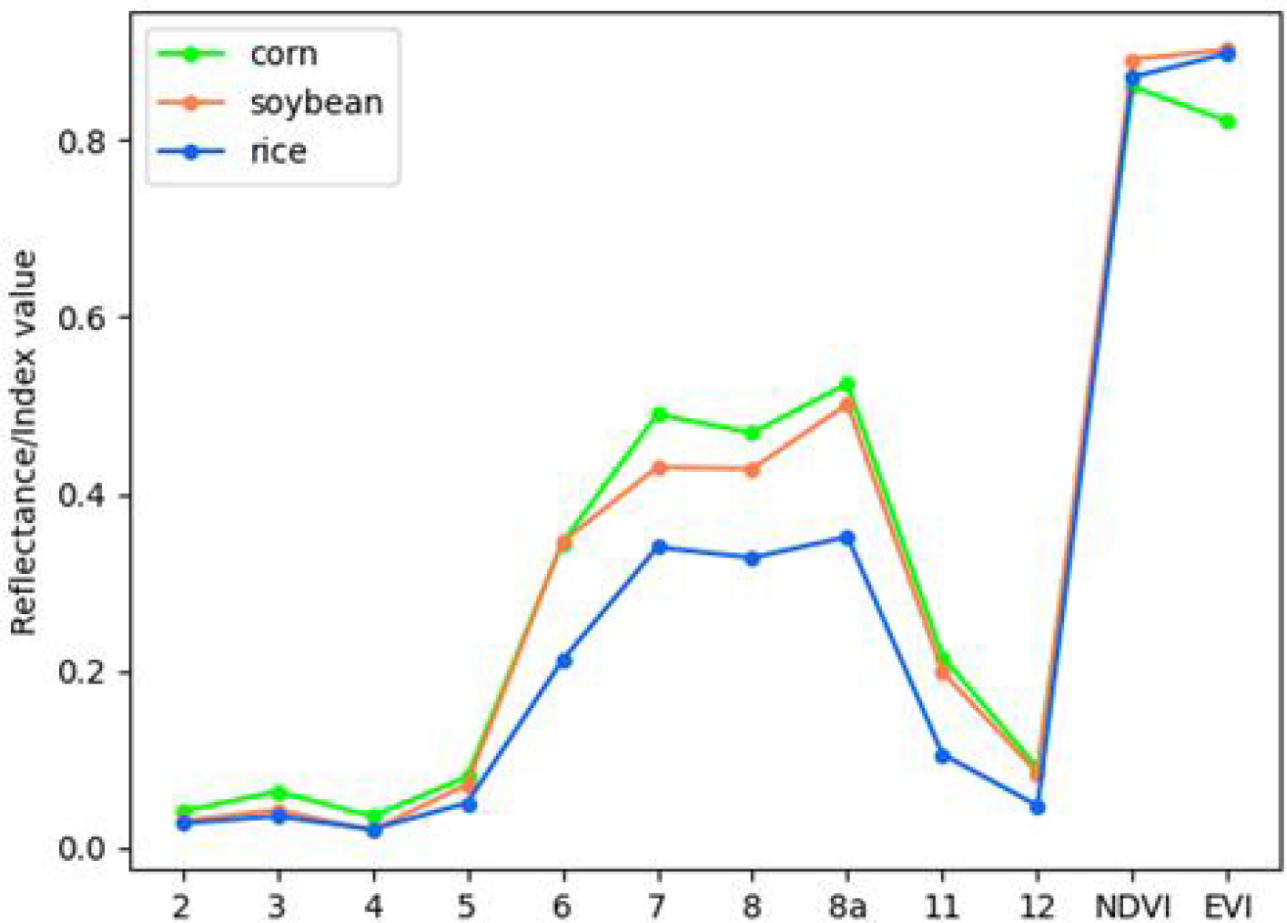
Mean reflectance spectral of each crop.

In this study, based on Sentinel-2 multispectral images, we designed 12 features (**Table 1**), including blue (band 2), green (band 3), red (band 4), visible light and near infrared (band 5-band 8a), short wave and infrared (band 11-band 12). The other two features are Normalized Differential Vegetation Index (NDVI) and Enhanced Vegetation Index (EVI). **Table 1** shows the calculation methods of the two planting cover index data. NIR in the formula is band 8. Among different vegetation indexes, NDVI and EVI are important measurement parameters of surface vegetation cover and vegetation growth (Immitzer et al., 2016; Huang et al., 2019), which have been proved to be helpful to improve the classification accuracy (Ferrant et al., 2017; Silveira et al., 2017; Belgiu and Csillik, 2018). The average reflectance spectra of each type of crop are shown in **Figure 7**. Principal Components Analysis (PCA) is used to extract the first three principal components of the image after principal component transformation as characteristic variables to participate in the classification.

**Table 1:** Features designed in this study.

Band	Description	Central wavelength(nm)	Spatial resolution(m)
band 2	Blue	490	10
band 3	Green	560	10
band 4	Red	665	10
band 5	Vegetation Red Edge	705	10
band 6	Vegetation Red Edge	740	10
band 7	Vegetation Red Edge	783	10
band 8	NIR	842	10
band 8a	Vegetation Red Edge	865	10
band 11	SWIR	1610	10
band 12	SWIR	2190	10
NDVI	$\frac{NIR-R}{NIR+R}$	–	10
EVI	$2.5*\frac{NIR-R}{NIR+6R-7.5B+1}$	–	10

**Figure 8:**
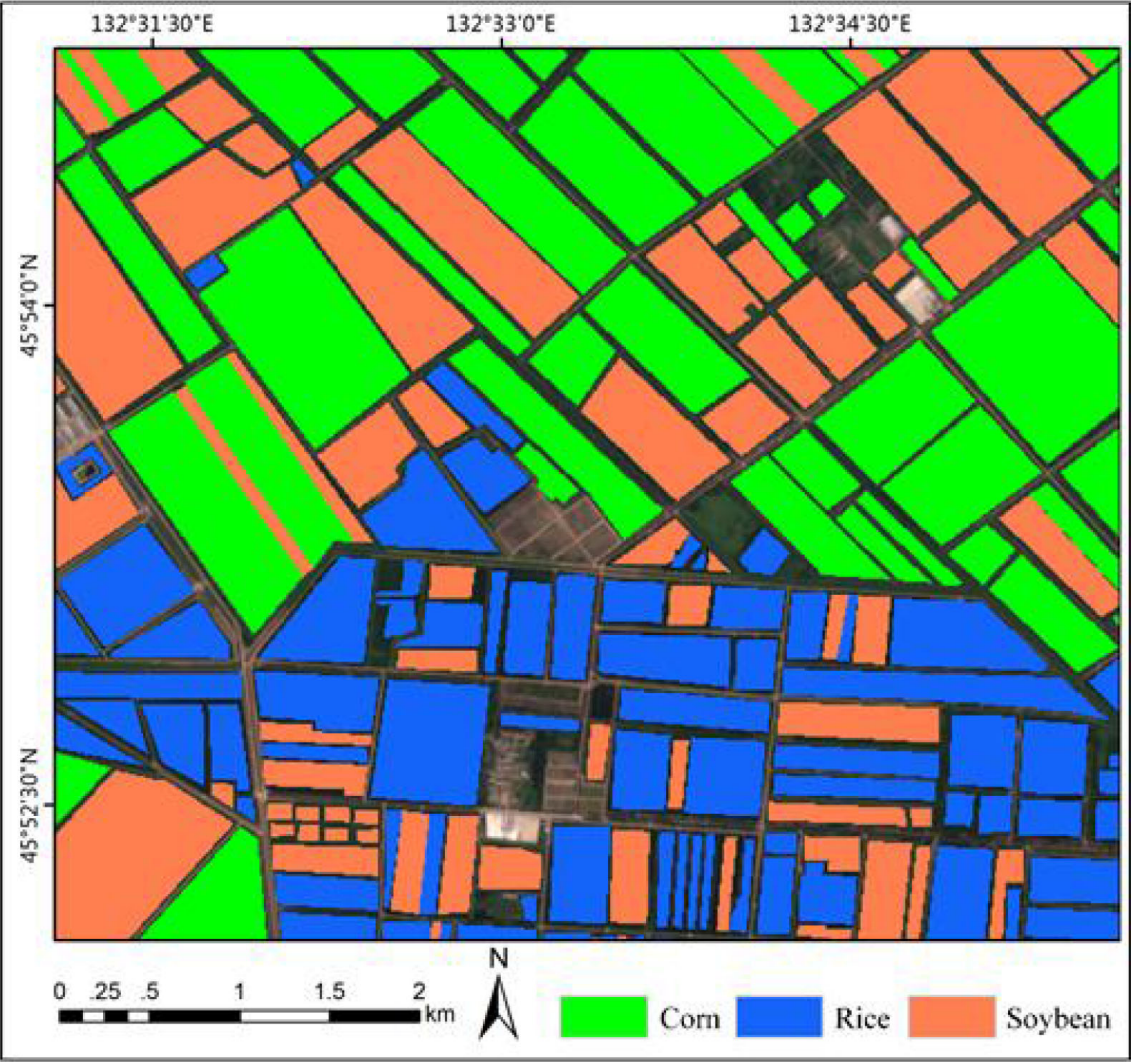
The ground truth used for model training and the effect of partial sample area zoomed.

The training set consists of 216 plots, including 50 plots of corn field, 86 plots of rice field and 80 plots of soybean field, as shown in **Figure 8**. The marks show the geographical locations of the real land cover sample areas extracted from the study area. The data of these plots were obtained through agricultural census and field survey, which collected a series of ground survey data. Including precise GPS coordinates of plots and crop types, pixel (sample) is the basic unit used for classification. **Table 2** lists crop types and the number of each type of sample in the training set.

**Table 2:** The number of training dataset per crop class.

Class	Label color	Samples size
Corn	▬	94842
Rice	▬	76964
Soybean	▬	82615

### 3.3 Comparison of backbone

**Figure 9:**
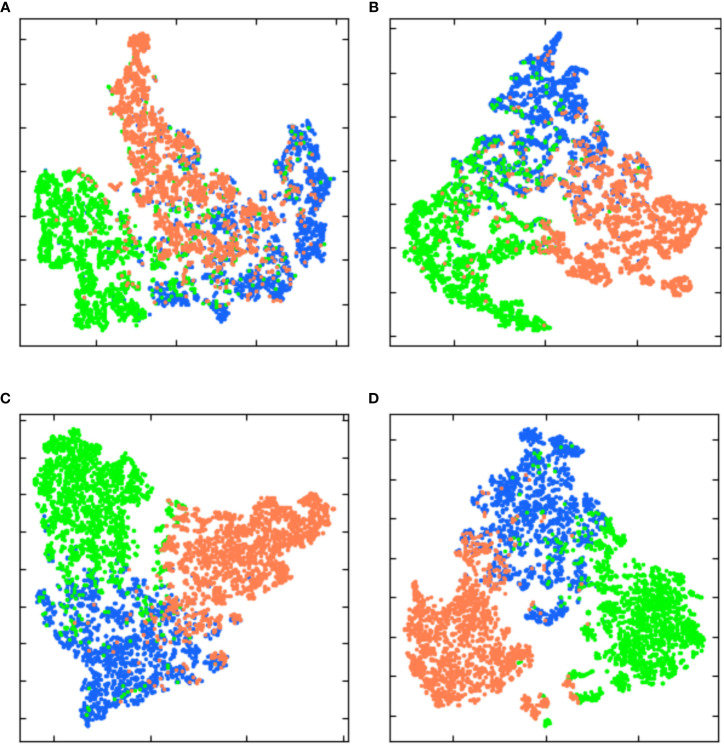
Two-dimensional plane projection of high-dimensional features learned by different backbone based on t-SNE. **(A)** original feature. **(B)** features extracted by ResNet18. **(C)** features extracted by VGG19. **(D)** features extracted by ResNet50.

Backbone is a model containing visual representation capability generated by pre-training upstream data, which is a part of deep learning model. Therefore, Backbone’s feature extraction capability directly affects the performance of the algorithm. This paper selects three different backbone networks, ResNet18, VGG19 and ResNet50, as feature extraction models of MSSNet. In the first experiment, T-distributed Random neighbor Embedding (t-SNE) was used to analyze the crop-specific spatial heterogeneity of the original Sentinel-2 data and the data processed by the deep learning model, so as to measure the feature extraction capability of different backbone. Then, 2000 samples each of crop are randomly selected, the original features and extracted features corresponding to these samples are nonlinearly projected to a 2-D plane for visualization using t-SNE. As shown in **Figure 9**, it is shown that the separability of features extracted by different backbone is significantly better than that of original features among different crop categories. In addition, compared with ResNet18 and VGG19, features extracted by ResNet50 are more separable and samples of the same crop category are more clustered. Therefore, ResNet50 is selected as the backbone network in this paper.

### 3.4 MSSNet architecture

**Figure 10:**
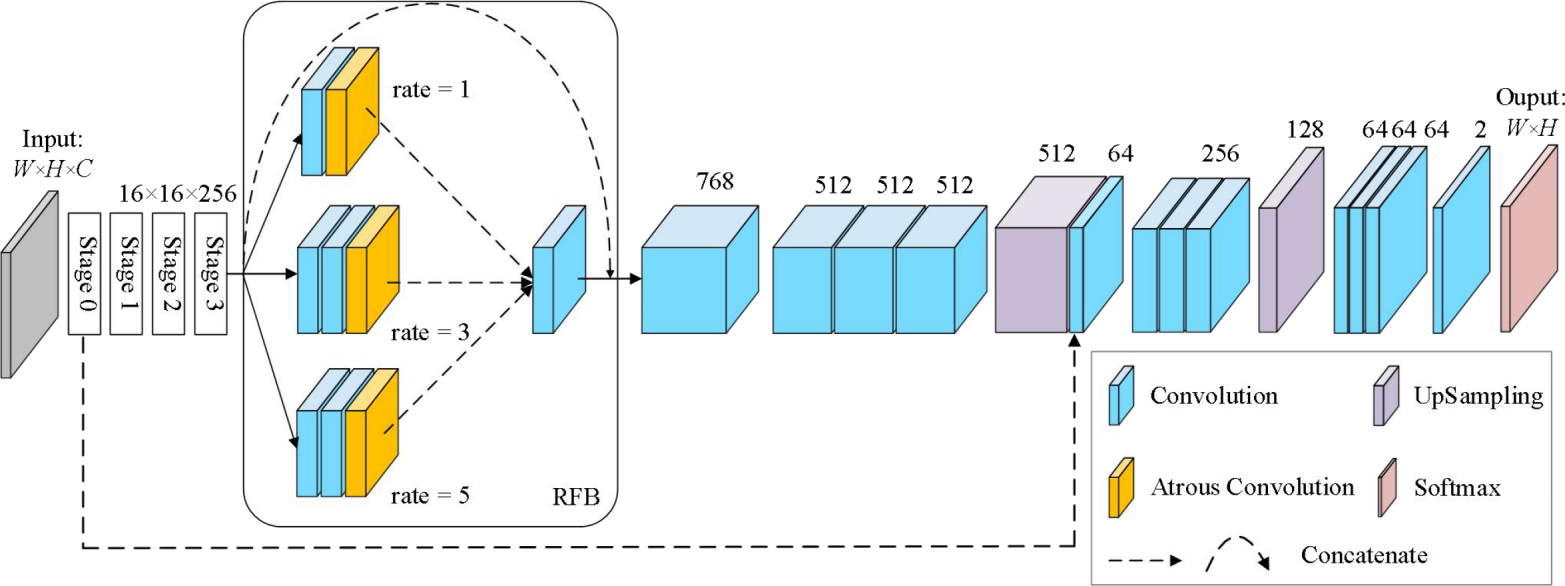
The architecture of MSSNet, ResNet50 and RFB are core components.

Inspired by the above multi-scale feature extraction modules, we proposed a semantic Segmentation network MSSNet (Multi Scale Segmentation Net) for fine classification of crops in agricultural areas. This network is based on residual network, receptive field module and skip connection. The architecture is shown in **Figure 10**, and the core contents are summarized as follows:

• The pre-trained residual network (ResNet50) is used as the backbone network to receive global visual features.
• Embedded receptive field module (RFB) for multi-scale feature extraction and integration.
• Use skip connection to concatenate low-level and high-level features with the same resolution.

The Input of MSSNet model is set as 256 × 256 × 3, and the backbone network ResNet50 is composed of 5 stages. We select the 39th convolutional layer (activation_39) located at Stage 3 as the output layer, and the size of the feature map is 16 × 16 × 256. The RFB module consists of four branches, and the identity mapping (arc) directly outputs 16 × 16 × 256. The second branch consists of a 3 × 3 conventional convolution and a dilated convolution with a dilation coefficient of 1, and the output feature map is 16 × 16 × 256. The third and fourth branches are both composed of two 3 × 3 conventional convolution and a dilated convolution. The dilation coefficients of the dilated convolution are different. Since the dilated convolution does not change the parameter number, the output of both branches is 16 × 16 × 256. RFB uses a 3 × 3 convolution to fuse the features extracted from the second to the fourth branches, and outputs the feature graph 16 × 16 × 768. At this time, the feature graph is added to the output of the identity map, and the final output of RFB is 16 × 16 × 768. After that, the feature dimension is reduced to 512, and the output feature graph is 16×16×512 for three consecutive 3 × 3 convolution. After the first quadruple up-sampling operation, the spatial resolution of the image is expanded to four times the original one, and the feature graph is 64 × 64 × 512. Considering the importance of course-scale features for semantic segmentation of fine-grained images, we fused low-level features with high-level features, and used a skip connection to achieve this in MSSNet. The convolutional layer activation_9 was located at Stage 0 of ResNet50, and its output was 64 × 64 × 64. The skip connection fuses 64 × 64 × 512 of the first up-sampling feature with 64 × 64 × 64 × 64 of the lower-level features, and outputs 64 × 64 × 576. After three 3 × 3 convolutions, the feature dimension is reduced to 256, and the spatial resolution of the image is restored to 256 × 256 by a second quad up-sampling. Finally, the probability that the output pixels of four 3 × 3 convolution layers and one convolutional layer using Softmax activation function belong to a certain class is obtained.

**Figure 11:**
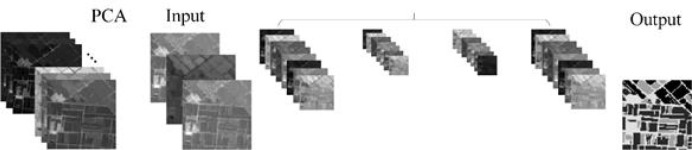
Semantic segmentation diagram of MSSNet, intermediate feature mapping represents features extracted at different levels.

In this paper, Python language is used to implement the MSSNet semantic segmentation network based on Keras API (Tensorflow as the back-end), and the network is used to mine spatial features and spectral features from multi-spectral data sets to achieve semantic segmentation. As shown in **Figure 11**, the reconstructed multispectral data was reduced from 12 features to 3 features by PCA, and then pixel-level classification results were output by ResNet50, receptive field module and continuous upsampling.

## 4 Experiment setting

### 4.1 Classification results and accuracy evaluation

**Figure 12:**
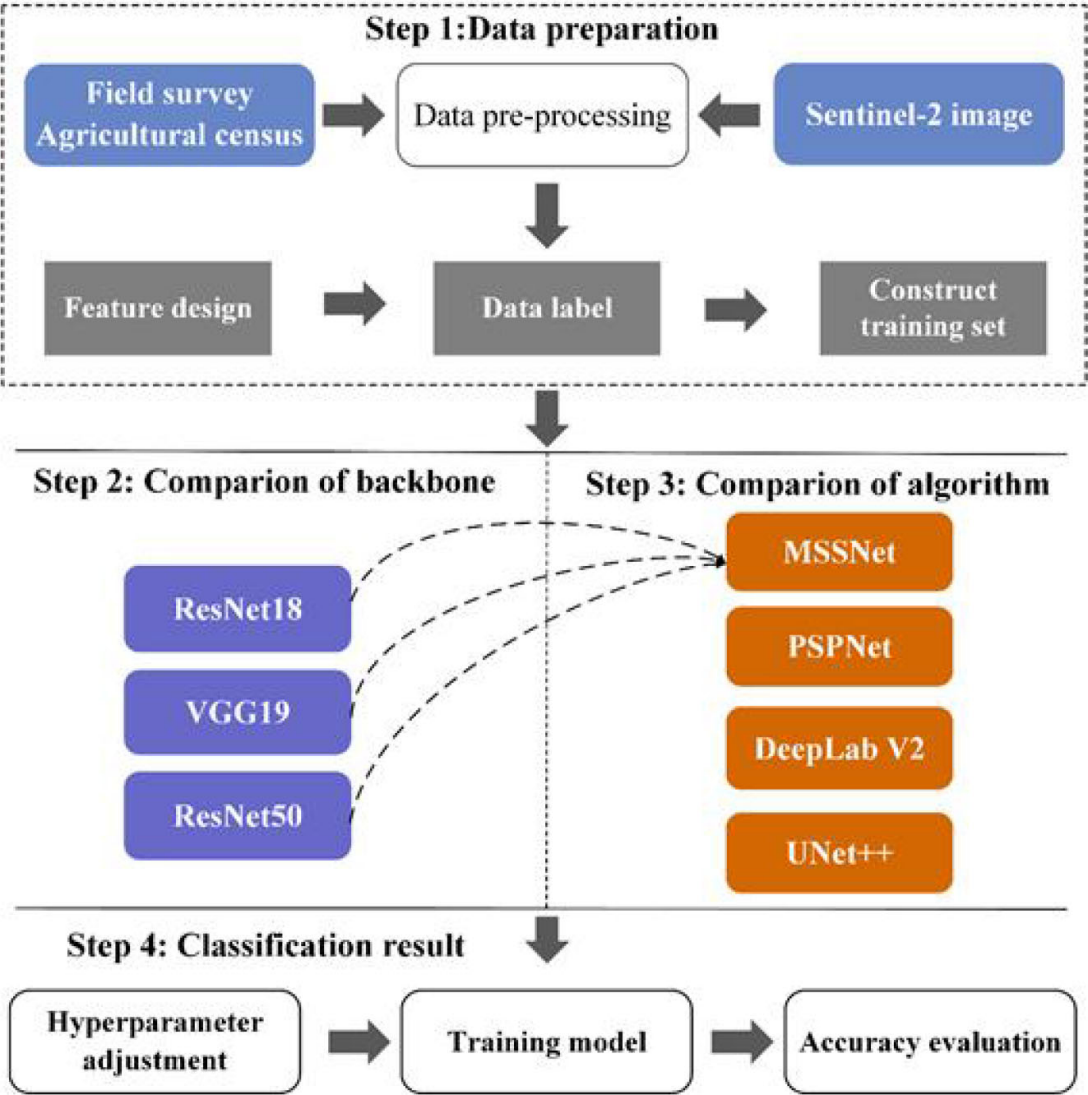
Experimental design.

In this paper, four deep learning semantic segmentation networks, including MSSNet, are applied to this classification task, and the experimental setup is shown in **Figure 12**. UNet++ is a deeply supervised semantic segmentation network where subnetworks of encoder and decoder are connected to each other through a series of nested dense jump paths, and PSPNet and DeepLab V2 are deep learning models for intensive prediction tasks. In the process of model training, the hyperparameters are also configured in the same configuration. The optimizer Adam has a learing rate of 0.001, iteration times (epoch) of 120, and batch size of 16. Input image resolution is set to 256 × 256, channel number *C* = 3, and is composed of the first three components after principal component transformation of Sentinel-2 image. Therefore, the input image size of the model is (256,256,3). In order to meet the architecture design of MSSNet, samples need to be extracted from image data. After rearrangement and normalization, the data enhancement strategy was used in the training process.

**Figure 13:**
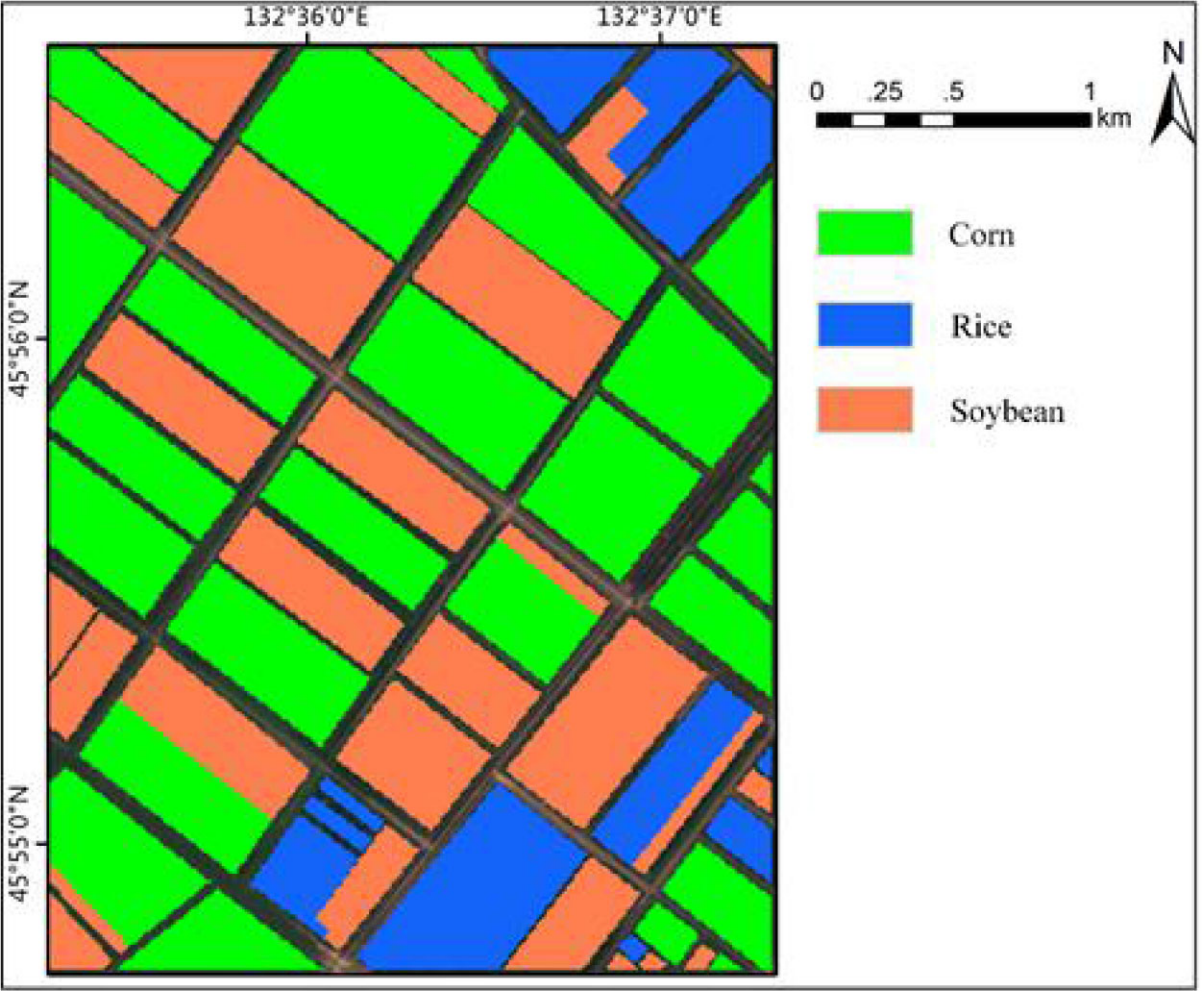
The test set is located in the study area and covers only one training sample.

In order to quantitatively and accurately assess the influence of different classifiers on crop extraction accuracy, an area of about 9 square kilometers in the research area is selected as the test set, as shown in **Figure 13**. The marked part is the test set, and the red box is the research area. 61 plots are marked in the test set, including 24 corn fields, 11 paddy fields and 26 soybean fields. The corresponding test samples are 35,368, 9711 and 24,933 respectively.

**Figure 14:**
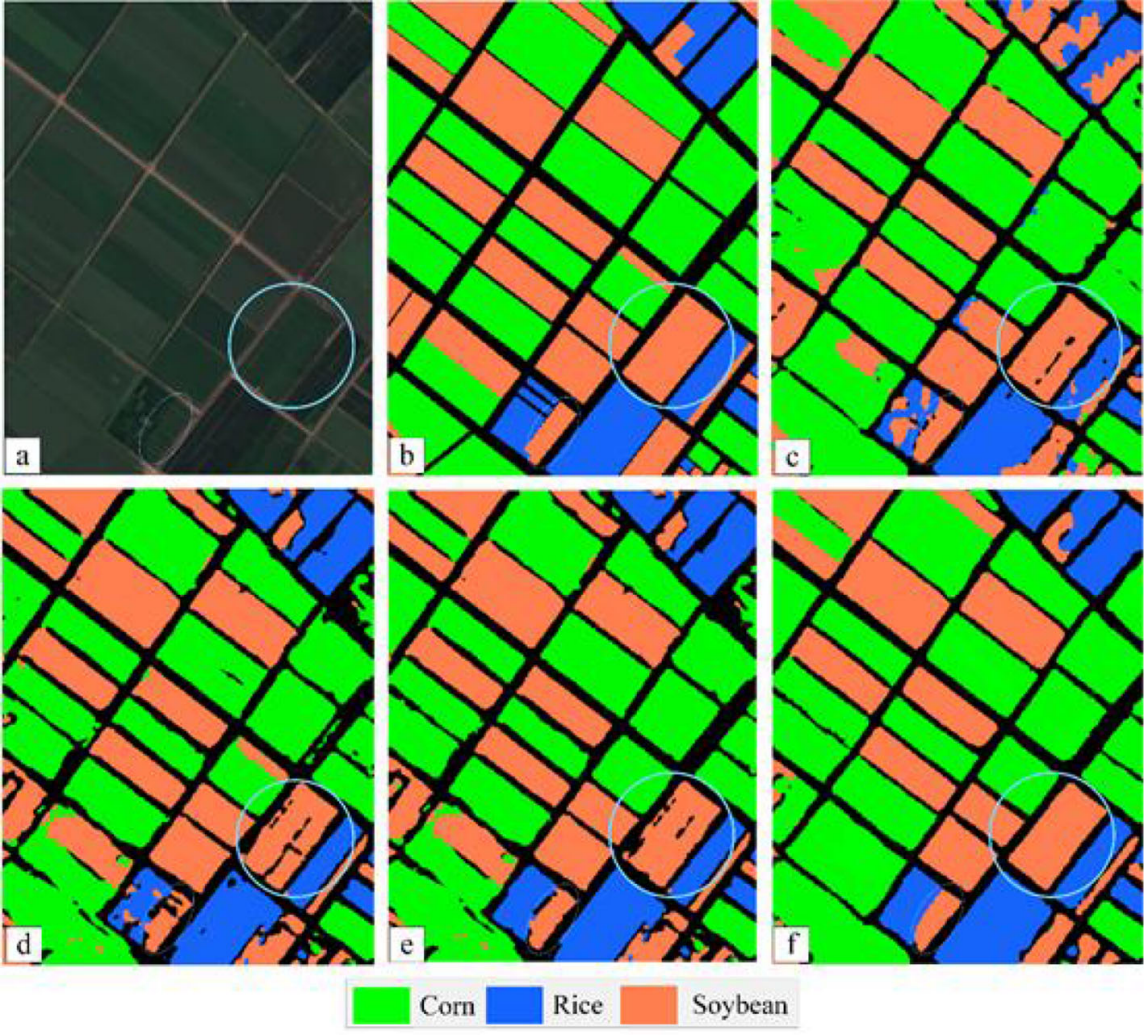
The segmentation result of each algorithm. **(A)** Sentinel-2 MSI image; **(B)** The ground truth; **(C)** UNet++; **(D)** PSPNet; **(E)** DeepLab V2; **(F)** MSSNet.

We evaluate the performance of the classifier using Mean Intersection over Union (MIoU), overall accuracy (OA), and the Kappa coefficient shown by the confusion matrix, which is the most commonly used metric for semantic segmentation tasks. MIoU calculates the IOU (the intersection of the real label and the predicted result) for each class separately, and then averages the IOU for all classes. MIoU is the standard accuracy measure. Among them, the overall accuracy can reflect the overall performance of the classifier. Each classification algorithm is trained five times repeatedly, that is, the same classification algorithm will make five predictions on the test set. The combined statistical results of the repeatedly generated confusion matrix are shown in **Table 3**. The crop classification diagram generated by different classification algorithms is shown in **Figure 14**.

**Table 3:** Classification accuracies of different algorithms, bold values show the best performance.

Class	UNet++ Mean ± SD	PSPNet Mean ± SD	DeepLab V2 Mean ± SD	MSSNet Mean ± SD
Corn	91.13 ± 0.69%	91.64 ± 1.26%	**92.55 ± 2.36%**	92.41 ± 1.68%
Rice	90.26 ± 1.42%	91.06 ± 1.88%	91.38 ± 2.35%	**91.58 ± 2.46%**
Soybean	80.41 ± 2.31%	86.44 ± 1.95%	79.28 ± 2.48%	**88.19 ± 4.30%**
OA (%)	82.68 ± 1.46	86.90 ± 1.15	89.56 ± 1.89	**90.77 ± 1.31**
MIoU×100	74.24 ± 0.60	74.78 ± 0.37	75.82 ± 2.91	**76.59 ± 0.21**
Kappa × 100	74.96 ± 2.16	81.13 ± 1.21	79.85 ± 2.78	**83.53 ± 2.29**

The bold values mean the highest classification accuracy.

Overall accuracy (OA) refers to the ratio between the total number of correctly classified samples of all categories and the total ground truth value. As can be seen from **Table 3**, among the four semantic segmentation algorithms, the multi-scale feature fusion network MSSNet proposed by us achieves higher classification accuracy. The overall accuracy is 8%, 3.87% and 1.21% higher than UNet++, PSPNet and DeepLab V2, respectively. The average classification accuracy of corn and rice reached 90%, but the classification accuracy of soybean was relatively low, and there was obvious misclassification between corn and soybean. MSSNet has the highest MIoU, which means that the model has the best segmentation for various categories, in addition, through qualitative analysis of the classification map, it can be seen that MSSNet is obviously superior to the other three algorithms in the detail characterization ability of image segmentation. The boundary of the block is clearer, the classification results of the block interior are more continuous (blue circular area in **Figure 14**, and it can extract the small block area more accurately (blue oval area in **Figure 14**). These performance gains are due to the multi-scale feature extraction and multi-level feature fusion capabilities of the multi-RFB module. Specifically, the convolutional kernel and cavity convolution of different sizes of the RFB module extract rich multi-scale features. The classification results of UNet++ and PSPNet are relatively rough and significantly weaker than the other two algorithms in terms of image details. There are large pixel blocks on the classification map, and the segmentation results cannot restore the details of the input image, which is also the main reason for their low classification accuracy.

### 4.2 Traditional machine learning classification algorithm

Traditional machine learning classifiers such as random forest (RF) and support vector machine (SVM) have been widely applied to classification tasks with their good performance. In this study, we compared four traditional machine learning classification algorithms on the same data set, which are RF, SVM, kernel SVM and XGBoost. RF adopts an integration algorithm with high accuracy and can maintain accuracy even if there is a large amount of missing data. Over-fitting does not occur easily owing to randomly selected samples’ characteristics and some features’ random extraction in the training process (Cutler et al., 2004). Support vector machine (SVM), first proposed by Corinna Cortes and Vapnik et al. in 1995, is a statistical theory specifically for small samples (Vapnik, 1995). Its unique advantage lies in dealing with small samples, nonlinear, and high-dimensional data problems, and many scholars have applied it to remote sensing image classification tasks.

**Figure 15:**
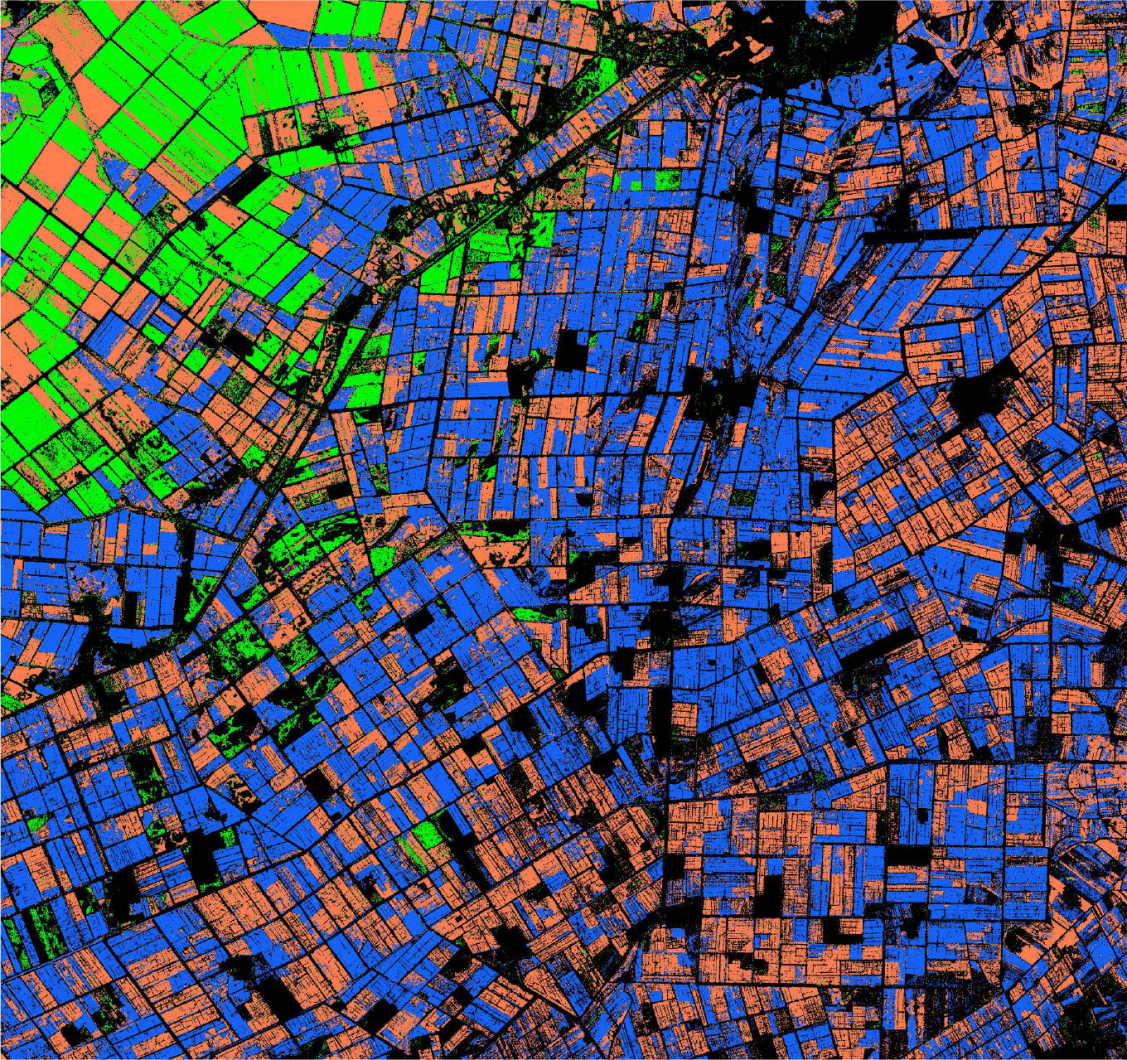
Crop classification in the study area using XGBoost algorithm.

**Figure 16:**
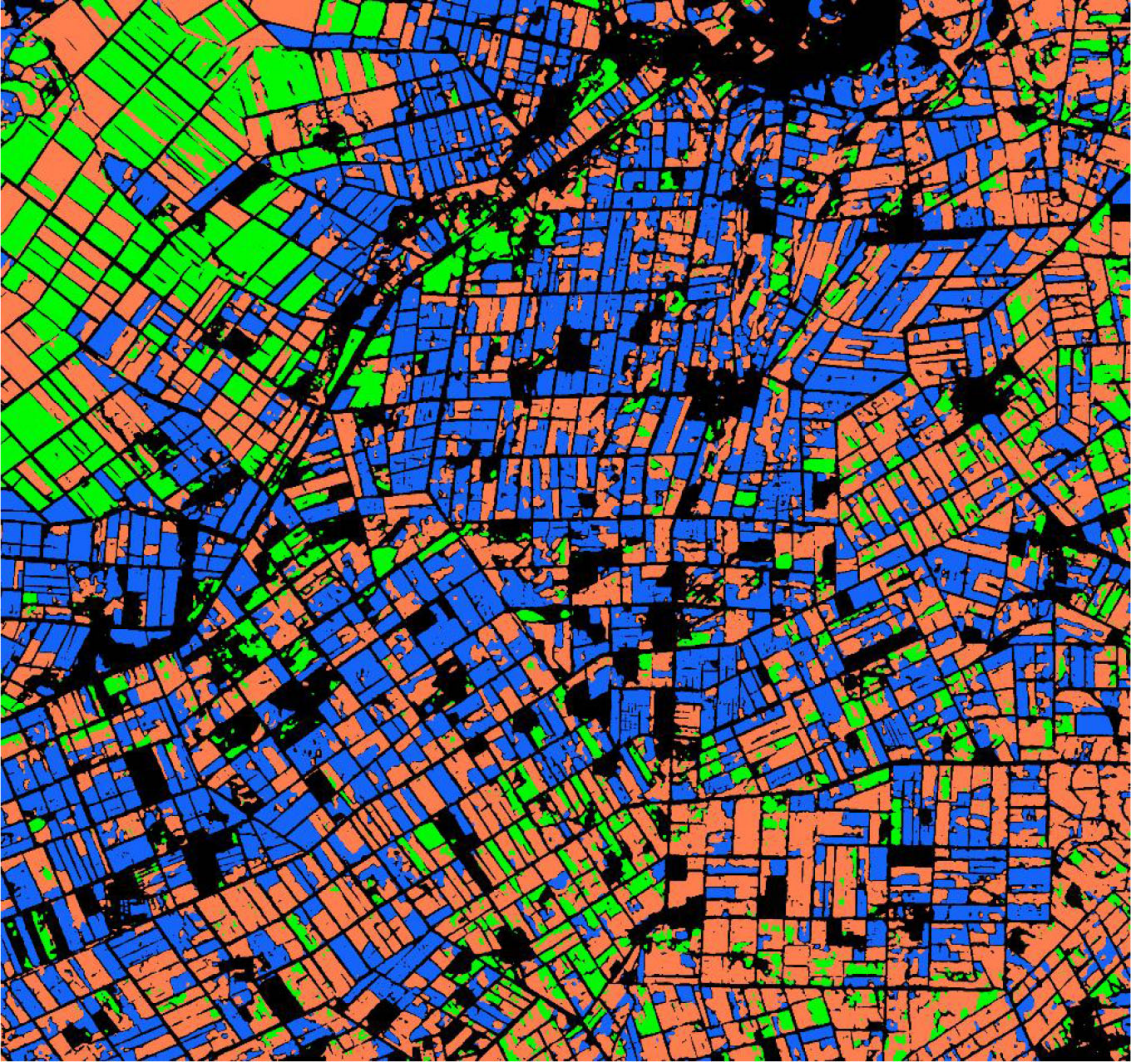
Crop classification in the study area using MSSNet model.

XGBoost is an open source machine learning project, which effectively implements GBDT algorithm with a lot of improvements, and has a wide range of applications in computer vision tasks such as image classification and object extraction. We use the “random search” method to optimize the main hyperparameters of the model, and select the best combination of hyperparameters from the candidate values according to the classification accuracy of each model on the test set. The optimization results are shown in **Table 4**. The bold characters in the candidate values represent the optimal parameters. Among these traditional machine learning classification algorithms, XGBoost has the best performance with 81.78% OA, Kernel SVM, RF and SVM 81.43%, 81.42% and 78.56%, respectively. According to the statistical data in **Tables 3**, **4**, the classification accuracy of the deep learning algorithm is better than that of the traditional machine learning algorithm on the whole. Even the classification accuracy of the rough FCN-32S model is slightly higher than that of XGBoost, while the highest classification accuracy of MSSNet is 90.68%, which is obviously higher than that of the traditional machine learning classifier. **Figure 15** and **Figure 16** show the crop classification in the study area of XGBoost and MSSNet, respectively.

**Table 4:** Comparison of traditional machine learning classifiers.

Classifier	Hyperparameters	OA	Kappa
Random Forest	n_estimators: 30, 50, 100, **200**, 300	81.42%	73.58%
	max_depth: 5, 10, **20**, 30, None		
	min_samples_split: **3**, 5, 10, 30, 100		
	min_samples_leaf: **1**, 3, 5, 7, 10		
SVM	C: 0.01, 0.05, 0.1, 0.5, **1**, 5, 10, 100	78.56%	69.55%
	Kernel: “linear”		
Kernel SVM	C: 0.01, 0.05, 0.1, 0.5, **1**, 5, 10, 100	81.43%	73.40%
	Kernel: “rbf”		
	Gamma:0.1, **0.2**, 0.3, 0.4, 0.5, 0.6, “auto”		
XGBoost	learning _rate: 0.01, **0.02**, 0.05, 0.1, 0.2	81.78%	74.07%
	gamma:0.05, **0.1**, 0.2, 0.5, 0.7, 1		
	max_depth:5, 7, 9, **15**, 17, 21, 25		
	min_child_weight:1, **5**, 7, 9, 11		
	subsamples:0.5, **0.6**, 0.8, 1		
	colsample_bytree:0.5, **0.6**, 0.8, 1		
	reg_labda:0.01, **0.1**, 1		
	reg_alpha:0, **0.**1, 0.3, 0.5, 1		
MSSNet	**-**	**90.68%**	**86.75%**

The bold values mean the highest classification accuracy.

## 5 Discussion and conclusion

Crop classification is the basis of large-scale crop acreage estimation. Currently, advanced Earth observation technology can identify the spatial distribution of crops on the plot scale. In this study, Sentinel-2 multi-spectral image with a single time phase and deep learning algorithm were used to make an attempt on the task of crop fine classification. In this paper, a multi-scale feature potential representation network MSSNet is proposed. Using ResNet50 as the backbone network, the network uses convolution kernel and void convolution of different sizes in the multi-scale feature module, which can frequently merge the features of different scale branches, and then learn more accurate feature maps to assist classification decision. In the experiment, we compared 4 traditional machine learning classification algorithms with 4 deep learning algorithms including MSSNet, and the classification results show that the deep learning algorithm has obvious advantages, especially the algorithm we proposed has obvious improvement compared with other commonly used classification algorithms.

Existing studies have shown that the best time for crop identification is between week 11 and 20 during the growing period (Xu et al., 2021), In this paper, Sentinel-2 images from the 14th week of crop growth were used to explore the application potential of single phase remote sensing in crop classification. Temporal, spectral and spatial characteristics are the basis of crop classification based on remote sensing technology. The method of crop extraction by using time series image has become an important method to extract crop planting structure by making full use of the characteristics of crop seasonal rhythm. However, it is often difficult to obtain image data of large range and long time series. In this study, a high classification accuracy is achieved by using single-phase optical images with only spectrum-space features. The selection of input features has an important impact on the performance of the model. Since NDVI and EVI can distinguish the phenological differences of different crops, these two artificial features have been introduced into crop classification experiments in large numbers. This practice was followed in feature design in this paper. 12 features such as blue, green, red, near-infrared band, normalized vegetation index and enhanced vegetation index were selected as key features for crop identification. The distribution of the importance of input features is closely related to the model structure, and the distinction of subtle differences between categories is the key to fine-grained image classification. Specifically, the feature extraction ability of the model for local spatial features determines the degree of refinement of classification results. In this paper, a multi-scale feature fusion module is designed in semantic segmentation model based on void convolution technology. By combining feature maps of different scales, the expression of ground object details is enhanced on the premise of ensuring classification accuracy. At the same time, in object detection and semantic segmentation tasks, model performance is highly dependent on features extracted by backbone. Therefore, we believe that it is very necessary to analyze input features when designing deep learning models.

## Data availability statement

The original contributions presented in the study are included in the article/supplementary material. Further inquiries can be directed to the corresponding author.

## Author contributions

TL wrote the draft of the manuscript. TL, LW contributed to data curation, analysis. MG contributed to manuscript revision. All authors contributed to the article and approved the submitted version.
